# Protective Factors Against Hypertension: A Retrospective Population-Based Analysis of Resilience in High-Risk Groups

**DOI:** 10.7759/cureus.86411

**Published:** 2025-06-20

**Authors:** Chekwube M Obianyo, Patra C Ezeamii, Mahfuza Akter, Jennifer C Mbonu, Anthonia N Njoku, Ebele Mary Nwosu, Okelue E Okobi

**Affiliations:** 1 Jiann-Ping Hsu College of Public Health, Georgia Southern University, Statesboro, USA; 2 Department of Medicine, Sylhet Muhammad Ataul Gani (MAG) Osmani Medical College, Sylhet, BGD; 3 Department of Social Work, Hennepin Healthcare, Minneapolis, USA; 4 Department of Medicine and Surgery, Crimean Federal University, Simferopol, RUS; 5 Spine Clinic, University of Texas (UT) Southwestern Medical Center, Frisco, USA; 6 Department of Family Medicine, Active Care Medical Centre, London, CAN; 7 Department of Family Medicine, IMG Research Academy and Consulting LLC, Homestead, USA; 8 Department of Family Medicine, Larkin Community Hospital Palm Springs Campus, Miami, USA; 9 Department of Family Medicine, Lakeside Medical Center, Belle Glade, USA

**Keywords:** dietary fiber, hdl, hypertension, nhanes, protective factors, resilience, sleep

## Abstract

Background

While risk factors for hypertension (HTN) are well-known, some high-risk individuals remain free of the condition, suggesting the presence of protective factors.

Objective

The objective of this study is to identify protective factors associated with resistance to hypertension among high-risk individuals using National Health and Nutrition Examination Survey (NHANES) data.

Methods

A retrospective, cross-sectional analysis was conducted using NHANES data from 2007 to 2018. Multivariable logistic regression was used to examine the links between hypertension and possible protective factors such as high-density lipoprotein (HDL) cholesterol, sleep duration, and dietary fiber intake (DFI) while taking into account age, gender, and race/ethnicity.

Results

Higher HDL cholesterol and longer sleep duration were significantly associated with reduced odds of hypertension. Dietary fiber intake was not independently associated after adjustment.

Conclusion

HDL cholesterol and adequate sleep appear to offer protective effects against hypertension. Further studies should include genetic markers, inflammatory biomarkers, and omega-3 fatty acids to better understand biological resilience to hypertension.

## Introduction

Hypertension (HTN) continues to be one of the most prevalent and preventable chronic health conditions affecting people worldwide and contributing significantly to cardiovascular morbidity and mortality. Lifestyle and behavioral factors such as high dietary sodium, high caloric intake, obesity, smoking, and physical inactivity have all been central to developing high blood pressure (BP) [1]. The risk factors paint decades worth of prevention and intervention. According to some authors, there are individuals with multiple risk factors who never develop hypertension over their lifespan, implying a distinct subset of resilient people who seem to biologically or behaviorally “protect” against the development of hypertension [2-4]. This population-based resilience has recently gained more attention as a potential gateway for finding novel protective factors to inform future clinical and public health strategies [5].

This study attempts to find protective factors that may confer some resistance to hypertension in such groups. Other scholars argue that, so far, the evidence suggests that dietary fiber intake (DFI) and omega-3 fatty acid intake, good sleep quality, good biomarkers, and even genetic variants may all play a role in such resilience [3]. It has been said that certain foods, especially omega-3 fatty acids, eicosapentaenoic acid (EPA) and docosahexaenoic acid (DHA), help improve blood vessel function and lower overall inflammation by affecting how blood vessels tighten and relax [6,7].

There has also been a considerable contribution of sleep quality and hygiene to cardiovascular health [1]. The risk factors of hypertension include chronic sleep deprivation (SD), irregular sleep patterns, and obstructive sleep disorders [1]. The third category of interest in the research is biomarkers that reflect underlying physiological mechanisms. For instance, low levels of C-reactive protein (CRP), which indicate systemic inflammation, along with higher levels of high-density lipoprotein (HDL) cholesterol (considered good cholesterol) and normal serum creatinine (indicating normal renal function), can serve as markers for general metabolic resilience [6]. In addition to indicating a lower burden of hypertension, these markers may also act as suppressors of other protective mechanisms that help prevent the development of hypertension.

There is genetic information regarding molecular-level protection [2]. Many specific changes in single-nucleotide polymorphisms (SNPs) in the *AGT*, *ACE*, *ADD1*, and *NOS3* genes are involved in controlling blood pressure through blood regulation processes [2]. For example, the *AGT M235T* polymorphism has been linked to lower angiotensin II activity, while certain *ACE* and *NOS3* variants enhance nitric oxide bioavailability, both mechanisms that help maintain vascular tone and sodium balance [2]. Other variants may lower angiotensin effects, boost nitric oxide production, or correct sodium balance, protecting them from hypertension risk in unfavorable settings or behaviors.

This study shifts the focus from vulnerability to resilience in hypertension research by using a retrospective, population-based analysis to evaluate and identify protective factors in a clearly defined “high-risk” normotensive population. Here, “high risk” refers to adults aged ≥18 years who, despite never being diagnosed with hypertension, have at least one established cardiometabolic risk factor, namely, obesity (BMI of ≥30 kg/m²), high dietary sodium intake (≥2,300 mg/day), or a first-degree family history of hypertension. The primary objective is to determine which factors (dietary fiber and omega‑3 intake, sleep duration, HDL cholesterol, and selected genetic variants) independently predict resistance to hypertension among these high‑risk but normotensive individuals, using nationally representative National Health and Nutrition Examination Survey (NHANES) data from 2007 to 2018.

## Materials and methods

This study utilized a retrospective, cross-sectional design based on data extracted from the National Health and Nutrition Examination Survey (NHANES), spanning six consecutive cycles from 2007 to 2018 [8]. NHANES is a nationally representative program conducted by the Centers for Disease Control and Prevention (CDC) that combines interviews, physical examinations, and laboratory tests to assess the health and nutritional status of adults and children in the United States.

This study utilized hypertension (HTN) as the primary outcome and identified dietary fiber intake, sleep duration, and high-density lipoprotein cholesterol as protective factors, as shown in Table 1.

**Table 1:** Variable descriptions Y/N: yes/no

Variable	Description	Type	Category
Hypertension (HTN)	The presence or absence of a self-reported hypertension diagnosis (Y/N)	Primary outcome	Primary outcome
Dietary fiber intake (DIF)	Reported in grams per day, based on average intake calculated from dietary recall data	Protective factor	Dietary intake
Sleep duration (sleep hours)	Self-reported number of hours of sleep obtained on average per night	Protective factor	Sleeping disorder
High-density lipoprotein (HDL) cholesterol	Laboratory-measured HDL cholesterol in mg/dL	Protective factor	Biomarker

Study population

Participants aged 18 years and older were eligible for inclusion. We excluded pregnant women, those with unreliable or incomplete dietary recalls (as defined by NHANES quality flags), and individuals with missing data on key covariates (age, sex, race/ethnicity, BMI, and smoking status). From the total NHANES dataset of 34,275 adults (2007-2018), 34,232 participants met these criteria and had complete information on self-reported hypertension diagnosis, dietary fiber intake, sleep duration, and laboratory-measured HDL cholesterol.

Within this analytic sample, the participants were categorized as “hypertensive” (n = 11,388) if they reported ever being told by a health professional that they had high blood pressure and as “non-hypertensive” (n = 22,844) otherwise. To establish the high-risk, normotensive subgroup for our resilience analysis, we further identified all non-hypertensive participants who had at least one cardiometabolic risk factor, specifically obesity (BMI ≥ 30 kg/m²), high dietary sodium intake (≥ 2,300 mg/day), or a first-degree family history of hypertension. This deliberate focus on a normotensive but high-risk cohort allows us to assess how candidate protective factors operate in those most vulnerable to incident hypertension.

We assessed potential confounding variables by age, sex, race/ethnicity, BMI, smoking status, and total caloric intake in all subsequent multivariable models.

Statistical data analysis

Descriptive statistics were used to summarize demographic and health characteristics. Continuous variables were reported as means with standard deviations, while categorical variables were presented as frequencies and percentages. Both bivariate analyses, t-tests and chi-square tests, were used to compare protective factor distributions between hypertensive and non-hypertensive individuals. We used multivariable logistic regression models to find out how protective factors relate to hypertension status while considering other factors such as age, gender, and race. Statistical significance was determined at the 5% (p < 0.05) level of significance. All analyses accounted for the complex survey design of NHANES using appropriate sample weights.

## Results

Descriptive and bivariate analysis

Table 2 presents the distribution of key protective factors and demographic variables by hypertension status. A total of 34,275 participants were included in the analysis, with individuals categorized as either hypertensive or non-hypertensive based on self-reported diagnosis.

**Table 2:** Comparison of demographic and protective health factors by hypertension (HTN) status using descriptive statistics, t-tests, and chi-square analyses

Variable	Non-HTN (n = 22,844)	HTN (n = 11,388)	T-test	Chi-square	P-value
HDL (mg/dL)	53.16 ± 15.56	51.79 ± 16.30	7.29	-	<0.001
Sleep hours	7.01 ± 2.89	6.89 ± 3.67	2.74	-	0.01
Dietary fiber (g)	16.83 ± 10.77	15.90 ± 9.96	7.66	-	<0.001
Age	45.91 ± 15.52	58.59 ± 15.46	-65.47	-	<0.001
Gender	-	-	-	0.02	0.90
Male	10,763 (66.77%)	5,357 (33.23%)	-	-	-
Female	12,081 (66.73%)	6,031 (33.27%)	-	-	-
Race	-	-	-	422.65	<0.001
Mexican American	2,322 (74.7%)	788 (25.3%)	-	-	-
Other Hispanic	1,525 (68.6%)	699 (31.4%)	-	-	-
Non-Hispanic White	5,176 (64.1%)	2,901 (35.9%)	-	-	-
Non-Hispanic Black	2,916 (57.9%)	2,122 (42.1%)	-	-	-
Non-Hispanic Asian	1,978 (77.4%)	578 (22.6%)	-	-	-
Other race/multirace	585 (68.4%)	270 (31.6%)	-	-	-

We used independent t-tests for continuous variables and chi-square tests for categorical variables to find meaningful differences between the two groups.

High-density lipoprotein (HDL) cholesterol

People without high blood pressure had a much higher average HDL level (53.16 ± 15.56 mg/dL) than those with high blood pressure (51.79 ± 16.30 mg/dL), and this difference was statistically significant (p < 0.001). This finding suggests a potential protective role of elevated HDL cholesterol against the development of hypertension.

Sleep duration

The average reported sleep duration was slightly higher among non-hypertensive participants (7.01 ± 2.89 hours) than in hypertensive participants (6.89 ± 3.67 hours), with a statistically significant difference (p = 0.01). Although the difference is modest, it supports the hypothesis that sufficient sleep may contribute to resilience against hypertension.

Dietary fiber intake

Mean dietary fiber intake was significantly greater among non-hypertensive individuals (16.83 ± 10.77 g/day) compared to their hypertensive counterparts (15.90 ± 9.96 g/day) (p < 0.001). This finding aligns with existing literature suggesting dietary fiber as a cardioprotective dietary component.

Age

As expected, people with high blood pressure were significantly older (58.59 ± 15.46 years) than those without high blood pressure (45.91 ± 15.52 years), with a very significant p-value (p < 0.001), showing a strong link between getting older and the risk of high blood pressure.

Gender

Gender distribution was similar between groups, with no statistically significant difference observed (p = 0.90). Females comprised a slightly higher proportion in both groups (non-hypertensive, 52.88%; hypertensive, 52.96%), indicating that gender was not a distinguishing factor in this sample.

Race/ethnicity

Race and ethnicity distributions differed significantly between hypertensive and non-hypertensive individuals (p < 0.001). Non-Hispanic Asians exhibited the highest proportion of non-hypertensive status (77.4%), followed by Mexican Americans (74.7%). Conversely, non-Hispanic Blacks had the highest proportion of hypertensive individuals (42.1%), consistent with known racial disparities in hypertension prevalence.

Multivariate logistic regression

To identify independent associations between protective factors and hypertension status, a multivariable logistic regression model was fitted, adjusting for age, gender, and race/ethnicity. The results are presented in Table 3.

**Table 3:** Multivariable logistic regression assessing independent associations between protective factors and hypertension status HDL, high-density lipoprotein; DFI, dietary fiber intake

Variable	OR	95% CI	P-value
HDL (mg/dL)	0.99	(0.98, 0.99)	<0.001
Sleep hours	0.98	(0.96, 1.00)	0.04
DFI (g)	1.00	(0.99, 1.00)	0.20
Age	1.05	(1.04, 1.05)	<0.001
Male	0.94	(0.86, 1.03)	0.20
Other Hispanic	1.19	(0.98, 1.45)	0.08
Non-Hispanic White	1.32	(1.13, 1.53)	<0.001
Non-Hispanic Black	2.04	(1.74, 2.40)	<0.001
Non-Hispanic Asian	0.79	(0.65, 0.97)	0.02
Other race/multirace	1.19	(0.89, 1.58)	0.25

From the multivariable logistic regression analysis, higher levels of high-density lipoprotein (HDL) cholesterol were significantly associated with reduced odds of hypertension. In simpler terms, for every 1 mg/dL increase in HDL, the chance of having hypertension goes down by 1% (OR = 0.99; 95% CI: 0.98, 0.99; p < 0.001), showing that HDL is a helpful marker for protection against hypertension. Similarly, sleep duration was inversely related to hypertension, with each additional hour of sleep linked to a 2% reduction in hypertension odds (OR = 0.98; 95% CI: 0.96, 1.00; p = 0.04), suggesting a modest yet statistically significant protective role of adequate sleep. On the other hand, dietary fiber intake (DFI) did not show a strong link to hypertension after accounting for other factors (OR = 1.00; 95% CI: 0.99, 1.00; p = 0.20), suggesting that its possible benefits might be less important when considering age and other variables.

As people get older, their chances of having hypertension increase, with each year adding a 5% higher risk (OR = 1.05; 95% CI: 1.04, 1.05; p < 0.001), which matches what we already know from studies. Gender, however, was not significantly associated with hypertension in the adjusted model (OR = 0.94; 95% CI: 0.86, 1.03; p = 0.20), suggesting that male sex does not confer a differential risk in this analysis. We observed several significant disparities regarding race and ethnicity. Compared to Mexican Americans (reference group), non-Hispanic Whites had 32% higher odds of hypertension (OR = 1.32; 95% CI: 1.13, 1.53; p < 0.001), and non-Hispanic Blacks had more than double the odds (OR = 2.04; 95% CI: 1.74, 2.40; p < 0.001). In contrast, non-Hispanic Asians exhibited significantly lower odds of hypertension (OR = 0.79; 95% CI: 0.65, 0.97; p = 0.02). Other Hispanic individuals and those categorized as other race/multirace did not show statistically significant differences compared to the reference group (p = 0.08 and p = 0.25, respectively).

## Discussion

This study used NHANES data from 2007 to 2018 to explore protective factors that may contribute to resilience against hypertension in high-risk adults. These initial findings suggest that higher HDL levels, longer sleep duration, and greater dietary fiber intake are associated with reduced prevalence of hypertension. Our analysis identified high-density lipoprotein (HDL) cholesterol and sleep duration as independent protective factors. Higher HDL levels were strongly connected to a lower chance of high blood pressure, backing up its well-known role in heart health by reducing inflammation and managing fats in the body. Similarly, longer sleep duration and good sleep hygiene practices were modestly but significantly linked to reduced hypertension risk, emphasizing the importance of both sleep quantity and consistent sleep habits in regulating blood pressure and autonomic function.

Although dietary fiber intake (DFI) showed significance in unadjusted comparisons, its effect was not independent after controlling for age and other variables. The finding suggests that its potential benefits may be mediated through other pathways or confounded by age-related dietary patterns. Age remained a strong predictor of hypertension, and notable racial disparities were observed, particularly among non-Hispanic Blacks, who had the highest odds of hypertension.

Still, similar to our study, other studies have increasingly emphasized the role of protective factors in resisting hypertension, particularly among high-risk individuals in the clinical setting [9]. The majority of research on hypertension has primarily centered on locating risk factors; however, more recent studies indicate that the existence of prevention methods may provide fresh perspectives into mechanisms of resilience [10]. Still, several empirical studies highlight the cardioprotective effects of HDL cholesterol, suggesting it plays an important role in lowering systemic inflammation and aiding endothelial function [11,12]. Further, comparable to our findings, sleep duration and sleep quality have also been linked to the regulation of blood pressure [13]. As an example, the analysis of NHANES data showed that participants with consistent and adequate sleep had lower systolic and diastolic pressure [13]. Furthermore, some dietary constituents, especially fiber and omega-3 fatty acids, while not consistently supported when controlled for confounding variables, do promote vascular health and reduce oxidative stress [14]. Other genetic studies provide additional evidence for the existence of protective single-nucleotide polymorphisms (SNPs) associated with angiotensin and nitric oxide synthesizing pathways [15]. Although these findings are encouraging, few investigations to date have tried to study these aspects within a single population [16].

These findings highlight that some individuals maintain normal blood pressure despite exposure to common risk factors. Understanding and promoting such protective factors, especially HDL maintenance and adequate sleep, may inform more effective, prevention-oriented public health strategies. Primary care providers may utilize these findings to guide customized counseling for individuals at high risk of HTN. There is a need for patients to be educated on lifestyle changes that result in HDL elevations, including healthy fats, aerobic exercises, sufficient sleep, and the reduction of refined carbohydrate consumption, while evaluating for and tackling sleep disorders that include obstructive sleep apnea and insomnia through effective interventions. The framing of such measures as evidence-based interventions to lower the risk of hypertension is likely to empower at-risk individuals to take proactive steps toward their cardiovascular health. Additionally, high uric acid levels can lead to hypertension because they trigger the renin-angiotensin system and cause problems with blood vessel function, while lower HDL cholesterol decreases the protection of blood vessels due to its anti-inflammatory effects.

Some other regulatory factors have also been recognized as leading factors that protect against HTN. The uric acid/HDL cholesterol ratio (UHR) is also recognized as a factor in hypertension, and Han and colleagues found that there is a positive link between UHR and hypertension in women who can have children [17]. Diet has also been implicated; a recent study disclosed that a vegetarian diet significantly reduces HTN risk. The vegetarian population, when matched for age and sex, has been found to have a 34% lower HTN risk compared to the non-vegetarian population [17,18]. Scholars have also studied other regulatory factors, including reflexes such as the baroreflex and the humoral regulator renin-angiotensin-aldosterone system (RAAS). Individuals with sleep deprivation (SD) often experience changes in BP control regulatory mechanisms, including baroreflex alterations, which result in the inability to lower BP at night [19]. The other mechanism entails the alteration of the sodium regulation systems. SD leads to the alteration of RAAS, nocturia, and natriuresis [20].

Study limitations and future directions

One key limitation of this study is its cross-sectional design, which precludes causal inference and the reliance on self-reported and variably defined measures (e.g., sleep duration and dietary sodium), which may introduce measurement error. The analysis also excludes other potential protective factors, including inflammatory markers such as C-reactive protein; kidney function indicators such as serum creatinine; genetic variants such as *AGT*, *ACE*, *ADD1*, and *NOS3;* and omega-3 fatty acids (EPA and DHA), due to the unavailability of data in the selected NHANES cycles. Unmeasured confounders, such as history of endocrine disorders, stress levels, menopausal status in women, and physical activity or lifestyle patterns, were not accounted for. Future studies should incorporate these variables and employ longitudinal designs to better establish causality and monitor protective effects over time.

## Conclusions

This study identified HDL cholesterol and sleep duration as independent protective factors against hypertension among US adults using nationally representative NHANES data from 2007 to 2018. By focusing on a clearly defined “high-risk” normotensive subgroup, those with obesity (BMI ≥ 30 kg/m²), high dietary sodium intake (≥ 2,300 mg/day), or a first-degree family history of hypertension, these findings emphasize the importance of shifting focus from solely identifying risk factors to understanding resilience mechanisms that may help prevent hypertension. Clinically, this suggests that interventions aimed at raising HDL and improving sleep quantity and hygiene could be prioritized in preventive counseling for individuals most vulnerable to developing hypertension. Public health strategies should similarly incorporate resilience-based messages alongside traditional risk-reduction approaches to more effectively curb hypertension incidence.
